# Anxiety and Depression among Hypertensive Outpatients in Afghanistan: A Cross-Sectional Study in Andkhoy City

**DOI:** 10.1155/2018/8560835

**Published:** 2018-08-01

**Authors:** Mohammad Shoaib Hamrah, Mohammad Hassan Hamrah, Hideki Ishii, Susumu Suzuki, Mohammad Hussain Hamrah, Ahmad Edris Hamrah, Ahmad Elias Dahi, Kyosuke Takeshita, Maimaiti Yisireyili, Mohammad Hashem Hamrah, Akbar Fotouhi, Junichi Sakamoto, Toyoaki Murohara

**Affiliations:** ^1^Department of Cardiology, Nagoya University Graduate School of Medicine, Nagoya, Japan; ^2^Dr. Mohammad Hashem Hamrah's Curative Clinic, Andkhoy, Afghanistan; ^3^Department of Clinical Laboratory, Nagoya University Hospital, Nagoya University, Graduate School of Medicine, Nagoya, Japan; ^4^Department of Epidemiology and Biostatistics, School of Public Health, Iran; ^5^Tokai Central Hospital, Kakamigahara, Japan

## Abstract

There is a relationship between mental and physical health. Depression and anxiety are linked with the development of several chronic diseases. The purpose of the present study was to determine the prevalence and factors associated with anxiety and depression among adult hypertensive outpatients in Afghanistan.* Methods*. Two hundred thirty-four consecutive hypertensive patients from December 2015 to August 2016 were recruited to complete the Hospital Anxiety and Depression Scale (HADS) questionnaire, which has scores for classifying the participants having anxiety and depression symptoms.* Results*. Of the total 234 patients, 81 (34.6%) were males and 153 (65.4%) were females. The mean age was 54.6 ± 12.7 for the hypertensive patients with anxiety and 63.8 ± 15.0 for the hypertensive patients with depression while this figure was 49.5 ± 10.2 for the adult participants in general population in Kabul city (Saeed, 2013). The prevalence of anxiety and depression (42.3% vs. 58.1%) among hypertensive persons is compared with the same mental disorders among Afghan refugees (39.3% vs. 22.1%) in Dalakee Refugee Camp (in Iran) (Hosseini Divkolaye and Burkle, 2017). Of the total participants, 99 had anxiety (42.3%), 136 had depression (58.1%), and 66 had (28.2%) comorbid anxiety-depression. Multivariate analysis was used. For anxiety age, female gender, smoking, diabetes mellitus, and 2 or more chronic diseases had a significant association. For depression, age and diabetes mellitus had a significant association, and for comorbid anxiety, depression, age, diabetes mellitus, and 2 or more chronic diseases had a significant association.* Conclusion*. This study shows that anxiety and depression are highly prevalent among hypertensive patients in an outpatient clinic in Afghanistan. There was an association between some sociodemographic and clinical characteristics and anxiety and depression. More studies are needed on a national level to inform the development of strategies for the prevention and control of psychological distress among patients with chronic diseases in Afghanistan.

## 1. Introduction

Noncommunicable diseases (NCDs) accounted for 71% (41 million) of a total of 57.7 million deaths globally. Of these, 80% (32.8 million) were attributed to cardiovascular diseases, diabetes, cancers, and chronic respiratory diseases [1]. About 80% of these NCD deaths (28 million) were recorded in low- and middle-income countries [2].

Globally, hypertension accounts for approximately 7.5 million deaths per year: nearly 12.8% of the total deaths [3]. In 2000, Kearney et al. estimated that 26.4% (972 million) of the world's population had hypertension [4]. The prevalence of hypertension is on the rise in low- and middle-income countries, whereas it has remained stable or fallen in developed countries [5].

In a study among males aged 15 years and older in Kabul city, the prevalence of smoking was 35.4% [6], while in a study to identify the prevalence and risk factors of NCDs among the older adult population (aged ≥ 40 years) in Kabul city in 2012, the prevalence of diabetes mellitus was reported to be 13.3%, obesity was 31.2%, and hypertension was 46.2% [7]. While previous studies have reported a high prevalence of NCDs risk factors in neighboring countries such as Pakistan and Iran [8–10], there is a paucity of information concerning the prevalence of risk factors for NCDs in Afghanistan. According to the results of a survey in 2015, Afghan people receive primary and secondary healthcare services through the Basic Package of Health Services and Essential Package of Hospital Services. However, these packages cover only 57% of the population [11]. In 2009, Amare et al. reported that about 60% of Afghan people obtained healthcare services via private hospitals and ambulatory care services [12].

Mental disorders are quite common in all the countries of the world and have a great impact on socioeconomic development and growth [13]. More than a quarter of the global population will develop a mental disorder at some point in their lives [14]. The Afghan population continues to face major stressors due to ongoing conflicts in different parts of their country [15]. In Afghanistan, presently, there are no provincial statistics to describe the prevalence of mental disorders. According to previous studies conducted at outpatient cardiac wards in Iran, the prevalence of anxiety was 5–10% and the prevalence of depression was 15–20% [16]. Patients with a chronic illness such as hypertension are at risk of developing mental illness, particularly anxiety and depression [17]. Previous studies have assessed this association. However, the findings have been inconsistent [18, 19]. Therefore, the aim of this study was to determine the prevalence and factors associated with anxiety and depression among hypertensive patients visiting an outpatient clinic in Andkhoy, Afghanistan.

## 2. Materials and Methods

This cross-sectional study was conducted in the outpatient clinic in Andkhoy, Afghanistan, from December 2015 to August 2016. Two hundred and thirty-four consecutive hypertensive patients were included in this study. The subjects were patients aged 18 years and above with currently treated or newly diagnosed arterial hypertension (defined as a seated systolic/diastolic blood pressure of 140/90 mm Hg at rest) [20] and self-reported use of antihypertensive drugs. Patients with physical and neurocognitive disorders and female patients who were pregnant were ineligible due to the likely presence of gestational hypertension which may resolve after delivery [21].

The patients were evaluated during a clinic visit, and the study protocol included an assessment of data obtained from each patient. A two-part questionnaire was used in this study. The first part included demographic characteristics, body mass index (BMI) (weight in kilograms divided by height in meters squared), blood pressure patterns, cardiovascular history and the presence of cardiovascular risk factors, comorbidity, and current drug therapy. Overweight and obesity were defined as a BMI of 25 to < 30 kg/m^2^ and BMI ≥ 30 kg/m^2^, respectively [22]. Diabetes mellitus: patients were with a fasting blood glucose level of ≥ 126 mg/dL, a random blood glucose level of ≥ 200 mg/dL, or self-reported use of antidiabetic medications [23]. Current smokers were defined as patients who have smoked at least 100 cigarettes in their lifetime and currently smoke cigarettes. Past smokers were defined as patients who had smoked at least 100 cigarettes in their lifetime but currently do not smoke. Nonsmokers were defined as patients who have never smoked a cigarette or had smoked fewer than 100 cigarettes in their lifetimes (but had stopped smoking at the time of the interview) [24]. Left ventricular hypertrophy was calculated from the patient's ECG based on the Sokolow-Lyon index [25].

The second part evaluated anxiety and depression by using the Hospital Anxiety and Depression Scale (HADS) questionnaire: a clinical scale used to assess anxiety among outpatients. Anxiety is defined as the subjective experience of apprehension or dread and its symptoms and signs, whereas depression is a mood disorder that causes a persistent feeling of sadness and loss of interest [26]. HADS contains 14 questions, seven concerning anxiety (HAD-A) and seven for depression (HAD-D). Scores range from 0 to 21 in each subscale and these items are scored on a 4-point Likert scale, and total scores are in the range of 0–42. The following is considered: the higher the score, the more severe the symptoms. The scores are classified as follows: normal (0–7), mild distress (8–12), moderate distress (11–14), and severe distress (15–21) [27]. A study showed that the HADS is a very effective tool for diagnosis of depression and anxiety symptoms in outpatients of a general hospital [28]. The Persian version of the HADS was used. The questionnaire was validated with Cronbach's alpha coefficients of 0.86 and 0.78 for HADS-D and HADS-A subscales, respectively [29]. The specificity and sensitivity were 0.78 and 0.9 on the HADS-A and 0.9 and 0.79 on the HADS-D [30]. The questionnaire was modified into the Dari language. Persian and Dari are mutually intelligible varieties of the same language: the Persian spoken in Iran has more French loanwords, whereas Dari has more English loanwords. Therefore, the language experts confirmed the equivalence of concept in the questionnaire. Moreover, the cultural validation has been made because we wanted to check appropriateness of wording and exclude the potential misinterpretation due to different ways of thinking. The interviews were conducted by trained doctors. Words were explained if there was need for more clarification about the concept of a question.

The data were entered onto each patient's case report form. Informed consent was obtained from all patients. The study was approved by the scientific review committee of Balkh Regional Hospital.

### 2.1. Statistical Analysis

Data are presented as mean ± standard deviation. Categorical variables are expressed as count and percentages. Continuous data were compared by using an independent samples t test. Categorical data were compared by means of *χ*^2^ test. Univariate and multivariate analyses were performed to determine significant factors associated with anxiety and depression among patients with hypertension. Odds ratios (OR) with 95% confidence intervals (CIs) were used for comparisons. A P value of < 0.05 was considered statistically significant. All analyses were performed using the SPSS 20.0 software package (SPSS, Chicago, IL).

## 3. Results

Of the total 234 study participants, 81 (34.6%) were males and 153 (65.4%) females. The male-to-female ratio among patients was 0.53, while that in a previous study was 0.85 [31]. More than half of the patients were aged > 60. Of the total participants, 99 had anxiety (42.3%), 136 had depression (58.1%), and 66 had (28.2%) comorbid anxiety-depression. The sociodemographic and clinical characteristics of all patients are summarized in Table 1.

Table 2 shows the association between sociodemographic and clinical characteristics and depression and anxiety among hypertensive participants. The mean age of patients with depression, anxiety, and comorbid anxiety-depression was 63.8 ± 15.0 years, 54.6 ± 12.7 years, and 55.7 ± 15.3, respectively. There was a high percentage of females (79.8%) with anxiety. Of the patients who were married, 87.5% had depression, 76.8% had anxiety, and 74.2% had comorbid anxiety-depression. Anxiety, depression, and comorbid anxiety-depression were more common among those over 60 than other age groups: 45.4%, 64.7%, and 45.4%, respectively. Unemployment was common among patients with anxiety (53.5%). Diabetes mellitus was associated with anxiety (40.4%, P= 0.006), depression (44.8%, P < 0.001), and comorbid anxiety-depression (56.1%, P < 0.001). A high smoking rate was observed in patients with anxiety (53.5%). Left ventricular hypertrophy was significantly associated with anxiety and comorbid anxiety-depression (P = 0.008 and P = 0.011, respectively). Significant association was seen between depression and stroke (P = 0.016).

Multivariate logistic regression analysis was developed for variables significantly associated with anxiety, depression, and comorbid depression-anxiety.  Table 3 indicates factors associated with anxiety among hypertensive patients. The odds of anxiety among patients aged older than 60 years were 5 times higher than among patients aged 18 to 39 years (95% CI 1.66–15.17, P = 0.004). Females were 4.25 times more likely to have anxiety (95% CI 2.07–8.73; P < 0.001). The odds of anxiety among smokers were 2.95 times greater than nonsmokers (95% CI 1.55–5.63, P = 0.001). Diabetic patients were 4.90 times more likely to report anxiety (95% CI 1.19–20.22; P = 0.028). Patients who had two or more comorbid diseases were 5.66 times more likely to report anxiety (95% CI 1.37–23.42; P = 0.017).

Table 4 presents factors associated with depression and comorbid depression-anxiety among hypertensive patients. Patients aged > 60 years were more likely to have depression disorder (OR = 4.04, 95% CI 2.07–7.89; P < 0.001) and comorbid depression-anxiety (OR = 3.50, 95% CI 1.37–8.97, P = 0.009). Diabetic hypertensive patients had a 22.74% (95% CI 2.69–192.36; P = 0.004) higher risk of depression in comparison to those with only hypertension. Those with diabetes were more than 10 times more likely to report comorbid depression-anxiety compared to those without comorbid diseases (95% CI 2.79–37.67; P < 0.001). Hypertensive patients with two or more comorbid diseases were approximately 4 times more likely to have comorbid depression-anxiety compared to patients with only hypertension (95% CI 1.10–14.25; P = 0.035).

## 4. Discussion

To our knowledge, this represents the first study in Afghanistan on the prevalence of anxiety, depression, and associated factors among hypertensive patients using the HADS questionnaire. Mental health services are limited in Afghanistan. There are a few mental health centers in the country, and those that do exist have limited capacity and low levels of coverage [15].

Anxiety and depression were found to be common among hypertensive patients in an outpatient clinic in the northern part of Afghanistan. Of the 234 hypertensive patients studied, 42.3% had anxiety disorders and 58.1% demonstrated depressive disorders, being higher than a previous study among patients in a cardiovascular outpatient clinic in Iran [16]. A similar prevalence of anxiety (38.4%) [32] and depression (60%) was reported among primary healthcare (PHC) patients [33]. The findings of our study are supported by the presence of a high prevalence of mental health problems in PHC patients with chronic diseases as reported by the World Health Organization and World Organization of Family Doctors [34].

The results of our study indicate that anxiety is significantly higher among older patients compared to younger patients. This finding is in accordance with a previous research conducted among type 2 diabetes outpatients in Malaysia [27]. A possible reason for this association might be that the incidence of cardiovascular disease, stroke, and cancer is a predictive factor for anxiety among older patients with chronic diseases [35].

Our study found that being a female is an independent risk factor for anxiety. This finding is comparable to the results of a previous study which was conducted in a PHC center in Al-Khobar city, Kingdom of Saudi Arabia [32]. This association may partly be explained by the fact that hormonal changes associated with the pregnancy, postpartum, and postmenopausal periods of women's lives have been linked to anxiety [36]. Another possible explanation for this is that Afghan women are more vulnerable to mental disorders due to a lack of mental health facilities and cultural issues [37].

In this study, smoking is an independent risk factor for anxiety. This association has been demonstrated in a previous survey [38]. In addition, it has been reported that individuals with increased anxiety are more likely to smoke [39]. This could be explained by a relevant study that found smoking both decreases anxiety and seems to increase the risk of developing increased anxiety [40]. Furthermore, patients with anxiety are more likely to have unhealthy behaviors, such as smoking and overeating [41].

We found a positive association between diabetes mellitus and anxiety among hypertensive patients. This association has been demonstrated in a study in PHC centers of the Supreme Council of Health, in Qatar [42]. It is believed that there has been a positive contribution of type 2 diabetes in increasing the occurrence of anxiety disorders in patients with hypertension [43].

There was a high rate of depression among older patients in this study, in agreement with a study from a hypertension outpatient clinic in Hong Kong [44]. The study observed that an increase in the prevalence of depression among older people in developing countries could be attributed to the paucity of mental healthcare services and facilities that prevents the early diagnoses and treatment of depression at younger age, as well as preventing mental health progression and controlling its severity among older people [45]. Other possible reasons might be their economic instability and delay of healthcare services due to cost, as well as their restricted access to healthcare. All of these conditions might increase the prevalence of depression among older participants.

According to the results of our study, patients with an increase in the number of chronic diseases were more likely to have comorbid depression-anxiety, which is similar to a study conducted on among patients visiting a chronic disease clinic in southwest Trinidad [46]. The fact that these results are due to patients with multiple chronic diseases feeling uncertain and worried about how their life is going to be with their illnesses seems possible. Sometimes, these worries and uncertainties are manifested as comorbid anxiety-depression [46].

Some limitations in the study should be discussed. First, the sample size consisted of a single center data source. The single center nature of this study may affect its generalizability to the entire population of Afghanistan. However, our outpatient clinic is a referral center in our province and the patient pool should be considered to be reasonably sizeable and representative, assuming that morbidity patterns do not differ significantly in practice in various parts of Afghanistan. The results are in agreement with other studies and should be beneficial to the accumulation of data regarding this issue. In addition, the cross-sectional nature of the study makes it difficult to find out the temporality between hypertension diagnosis and mental health outcomes. Finally, the sample was nonrandom, thus limiting the overall power.

In conclusion, the prevalence of anxiety and depression among outpatients with hypertension was high. Anxiety and depression were significantly associated with some sociodemographic and clinical characteristics of patients. Therefore, more researches are needed on national level to develop strategies for the prevention and control of psychological distress among patients with chronic diseases in Afghanistan.

## Figures and Tables

**Table 1:** Sociodemographic and clinical characteristics of study participants.

Characteristics	Frequency (n=234)	Percentage (%)
Age		
<39	25	10.7
40-60	86	36.7
>60	123	52.5
Gender		
Male	81	34.6
Female	153	65.4
Level of education		
Illiterate	146	62.4
Primary/private education	17	7.3
Secondary	38	16.2
High school/ more	33	14.1
Marital Status		
Married	201	85.9
Single	24	10.3
Others	9	3.8
Occupation		
Employed	58	24.8
Unemployed	94	40.2
Housewife	50	21.4
Others	32	13.7
Smoking status		
Yes	94	40.2
No	140	59.8
Body mass index		
less than 25	129	55.1
25≥	105	44.9
Blood pressure control		
Yes	46	19.7
No	188	80.3
Diabetes mellitus	72	30.8
Left ventricular hypertrophy	130	55.6
Heart failure	30	12.8
Stroke	12	5.1
Myocardial infarction	44	18.8
Anxiety	94	40.2
Depression	136	58.1
Comorbid depression-anxiety	66	28.2

**Table 2:** Association between sociodemographic and clinical characteristics with depression, anxiety, and comorbid anxiety-depression among hypertensive participants.

	Depression		Anxiety		Comorbid depression-anxiety	
	n=136	P* value*	n=99	P* value*	n=66	P* value*
Age, mean, years mean (SD)	63.8±15.0	0.915	54.6±12.7	0.019	55.7±15.3	0.04
Gender		0.099		<0.001		0.573
Male,%	83 (61.0%)		79 (79.8%)		45 (68.2%)	
Female, %	53 (39.0%)		20 (20.2%)		21 (31.8%)	
Marital status		0.395		0.003		0.005
Married	119 (87.5%)		76 (76.8%)		49 (74.2%)	
Single	11 (8.1%)		17 (17.2%)		13 (19.6%)	
Others	6 (4.4%)		6 (6.0%)		4 (6.1%)	
Age (in years)		<0.001		0.001		0.019
≤39	12 (8.8%)		18 (18.2%)		13 (19.6%)	
40-69	36 (26.5%)		36 (36.4%)		23 (34.8%)	
>60	88 (64.7%)		45 (45.4%)		30 (45.4%)	
Level of education		0.193		0.414		0.026
Illiterate	85 (62.5%)		58 (58.6%)		33 (50.0%)	
Primary/private education	8 (5.9%)		6 (6.0%)		5 (7.6%)	
Secondary	27 (19.8%)		17 (17.2%)		18 (27.3%)	
High school/higher	16 (11.8%)		18 (18.2%)		10 (15.1%)	
Occupation		0.086		<0.001		0.075
Employed	30 (22.1%)		12 (12.1%)		9 (13.6%)	
Unemployed	54 (39.7%)		53 (53.5%)		33 (50.0%)	
Housewife	27 (19.8%)		25 (25.2%)		15 (22.7%)	
Others	25 (18.4%)		9 (9.1%)		9 (13.6%)	
Body mass index, mean (SD)	24.5±4.4	0.838	23.3±4.24	0.016	23.7±4.4	0.545
Diabetes mellitus	61 (44.8%)	<0.001	40 (40.4%)	0.006	37 (56.1%)	<0.001
Smoking		0.128		<0.001		0.055
Yes	49 (36.0%)		53 (53.5%)		33 (50.0%)	
No	87 (64.0%)		46 (46.5%)		33 (50.0%)	
Left ventricular hypertrophy	80 (58.8%)	0.236	45 (45.4%)	0.008	28 (42.4%)	0.011
Heart failure	22 (16.2%)	0.070	11 (11.1%)	0.503	8 (12.1%)	0.841
Stroke	11 (8.1%)	0.016	3 (3.0%)	0.213	3 (4.5%)	0.800
Myocardial infarction	34 (25.0%)	0.004	13 (13.1%)	0.057	12 (18.2%)	0.879

**Table 3:** Associated factors with anxiety among hypertensive patients.

Variables	Anxiety n (%)	OR	(95% CI)	P* value*
Age ≤ 39	18 (18.2)	1.0		
40-60	36 (36.4)	2.61	(0.866-7.88)	0.088
>60	45 (45.4)	5.0	(1.66-15.17)	0.004
Occupation, Employed	12 (12.1)	1.0		
Housewife	25 (25.2)	1.96	(0.62-6.24)	0.253
Others	9 (9.1)	0.287	(0.106-0.77)	0.014
Unemployed	53 (53.5)	0.431	(0.148-1.255)	0.123
Gender, Male	20 (20.2)	1.0		
Female	79 (79.8)	4.25	(2.07-8.73)	<0.001
Smoking, No	46 (46.5)	1.0		
Yes	53 (53.5)	2.95	(1.55-5.63)	0.001
Comorbidities				
Only hypertension	1.0			
with DM	40 (40.4)	4.90	(1.19-20.22)	0.028
DM with MI or Stroke	9 (9.1)	5.66	(1.37-23.42)	0.017

CI, confidence interval; OR, odds ratio; DM, diabetes mellitus; MI, myocardial infarction.

**Table 4:** Associated factors with depression and comorbidity of depression and anxiety among hypertensive patients.

	Depression			Comorbid depression-anxiety		
Variables	OR	95% CI	P* value*	OR	95% CI	P* value*
Age ≤ 39	1.0					
40-60	3.99	(1.41-11.28)	0.009	2.63	(1.00-6.94)	0.05
>60	4.04	(2.07-7.89)	<0.001	3.50	(1.37-8.97)	0.009
Comorbidities						
only hypertension	1.0					
with DM	22.74	(2.69-192.36)	0.004	10.24	(2.79-37.67)	<0.001
DM and MI or stroke	2.91	(0.34-25.03)	0.331	3.96	(1.10-14.25)	0.035

CI, confidence interval; OR, odds ratio; DM, diabetes mellitus; MI, myocardial infarction.
